# The Performance of a Dual-Energy CT Derived Radiomics Model in Differentiating Serosal Invasion for Advanced Gastric Cancer Patients After Neoadjuvant Chemotherapy: Iodine Map Combined With 120-kV Equivalent Mixed Images

**DOI:** 10.3389/fonc.2020.562945

**Published:** 2021-01-11

**Authors:** Lingyun Wang, Yang Zhang, Yong Chen, Jingwen Tan, Lan Wang, Jun Zhang, Chunxue Yang, Qianchen Ma, Yingqian Ge, Zhihan Xu, Zilai Pan, Lianjun Du, Fuhua Yan, Weiwu Yao, Huan Zhang

**Affiliations:** ^1^ Department of Radiology, Ruijin Hospital, Shanghai Jiao Tong University School of Medicine, Shanghai, China; ^2^ Department of Radiology, Zhejiang Provincial People’s Hospital, People’s Hospital of Hangzhou Medical College, Hangzhou, China; ^3^ Department of Oncology, Ruijin Hospital, Shanghai Jiao Tong University School of Medicine, Shanghai, China; ^4^ Department of Pathology, Ruijin Hospital, Shanghai Jiao Tong University School of Medicine, Shanghai, China; ^5^ CHN DI CT Collaboration, Siemens Healthineers Ltd, Shanghai, China; ^6^ Department of Radiology, Ruijin Hospital North, Shanghai Jiao Tong University School of Medicine, Shanghai, China; ^7^ Department of Radiology, Tongren Hospital, Shanghai Jiao Tong University School of Medicine, Shanghai, China

**Keywords:** locally advanced gastric cancer, dual energy CT, iodine map, radiomics, neoadjuvant chemotherapy

## Abstract

**Objectives:**

The aim was to determine whether the dual-energy CT radiomics model derived from an iodine map (IM) has incremental diagnostic value for the model based on 120-kV equivalent mixed images (120 kVp) in preoperative restaging of serosal invasion with locally advanced gastric cancer (LAGC) after neoadjuvant chemotherapy (NAC).

**Methods:**

A total of 155 patients (110 in the training cohort and 45 in the testing cohort) with LAGC who had standard NAC before surgery were retrospectively enrolled. All CT images were analyzed by two radiologists for manual classification. Volumes of interests (VOIs) were delineated semi-automatically, and 1,226 radiomics features were extracted from every segmented lesion in both IM and 120 kVp images, respectively. Spearman’s correlation analysis and the least absolute shrinkage and selection operator (LASSO) penalized logistic regression were implemented for filtering unstable and redundant features and screening out vital features. Two predictive models (120 kVp and IM-120 kVp) based on 120 kVp selected features only and 120 kVp combined with IM selected features were established by multivariate logistic regression analysis. We then build a combination model (ComModel) developed with IM-120 kVp signature and ycT. The performance of these three models and manual classification were evaluated and compared.

**Result:**

Three radiomics models showed great predictive accuracy and performance in both the training and testing cohorts (ComModel: AUC: training, 0.953, testing, 0.914; IM-120 kVp: AUC: training, 0.953, testing, 0.879; 120 kVp: AUC: training, 0.940, testing, 0.831). All these models showed higher diagnostic accuracy (ComModel: 88.9%, IM-120 kVp: 84.4%, 120 kVp: 80.0%) than manual classification (68.9%) in the testing group. ComModel and IM-120 kVp model had better performances than manual classification both in the training (both p<0.001) and testing cohorts (p<0.001 and p=0.034, respectively).

**Conclusions:**

Dual-energy CT-based radiomics models demonstrated convincible diagnostic performance in differentiating serosal invasion in preoperative restaging for LAGC. The radiomics features derived from IM showed great potential for improving the diagnostic capability.

## Introduction

Stomach cancer remains prevalent worldwide. There were over 1,000,000 new cases in 2018 from the disease, which resulted in an estimated 783,000 deaths (1 in every 12 deaths globally), making it the fifth most frequently diagnosed cancer and the third leading cause of cancer death. The incidence of stomach cancer in east Asia, particularly in China, is much higher than in any other region of the world (1). The high mortality rate is largely due to late diagnosis at locally advanced gastric cancer(LAGC) (2).Neoadjuvant chemotherapy(NAC) has been shown to significantly increase the curative resection rate, disease-free survival, and overall survival from this disease (3, 4). Serosal invasion and lymph node status after NAC were established to be independent prognostic factors (5, 6).

Endoscopic ultrasound (EUS) and computed tomography (CT) are the most frequently used methods for preoperative staging of gastric cancer and the accuracy varies among different studies: 78%–92% and 77%–89% for T staging and 57%–91% and 71%–90% for N staging for EUS and CT respectively (7–10). However, the accuracy of T and N restaging after NAC decreased to 47% and 39% by EUS, and to 57% and 37% by CT, respectively (11, 12). Compared with primary staging, restaging after NAC has been shown to be inaccurate and unreliable. The radiologic T stages were not significantly correlated with pathologic T stages, whereas the radiologic N and pathologic N stages were significantly correlated (12). At present, no diagnostic modality has been accepted as an effective method for restaging, particularly in T-restaging, which was once regarded as too weak for clinical decision-making. The accurate assessment of clinical T-restaging, particularly with the invasion of serosa after NAC, is critical for operative decision-making, as well as to evaluate prognosis. Therefore, improving the accuracy of restaging of serosal invasion after NAC is particularly critical.

Dual-energy CT (DECT) emerged as a cutting-edge technique that embraced a material decomposition algorithm (13, 14) to separate different materials and obtain quantitative material concentrations, such as iodine uptake (IU, mg/ml). Preliminary studies have reported the use of the IU in different tumors (15–17). A study in 2015 reported the utility of IU in evaluating the response after NAC in gastric cancer (14). However, this study only enrolled 20 patients, and the regions of interests (ROIs) were traced in round or oval shapes, which indicated that the diagnostic value of DECT had not yet been fully evaluated. In addition, there exist no related studies that assess the capacity of IU for restaging after chemotherapy in LAGC patients.

Radiomics extracts high-throughput quantitative imaging features and can characterize the spatial relationships and consistency of signal intensities within the tumor region. It has demonstrated the ability to predict treatment response or prognosis across a range of cancer types and imaging modalities, such as hepatocellular carcinoma, rectal cancer, breast cancer, and prostate cancer (18–20). By extracting image features such as shape, size, texture, and density, the images were then transformed into mineable high-dimensional data which improved medical decision-making and personalized precision medicine (19, 21). In addition, radiomics has shown its superiority in diagnosing lymph node metastasis and occult peritoneal metastasis (22, 23). However, the diagnosis value of radiomics, particularly combined with dual-energy technology in gastric cancer patients after NAC, remains unclear.

Our aim in this study was to explore the prediction performance of dual-energy CT-derived radiomics models and the incremental diagnostic value of IM features in preoperative restaging of serosal invasion with LAGC after NAC.

## Materials and Methods

### Patients

This study was approved by our Ethical Committee, and informed consent was waived for the patients. All procedures involving human participants adhered to the tenets of the Declaration of Helsinki.

**Figure 1:**
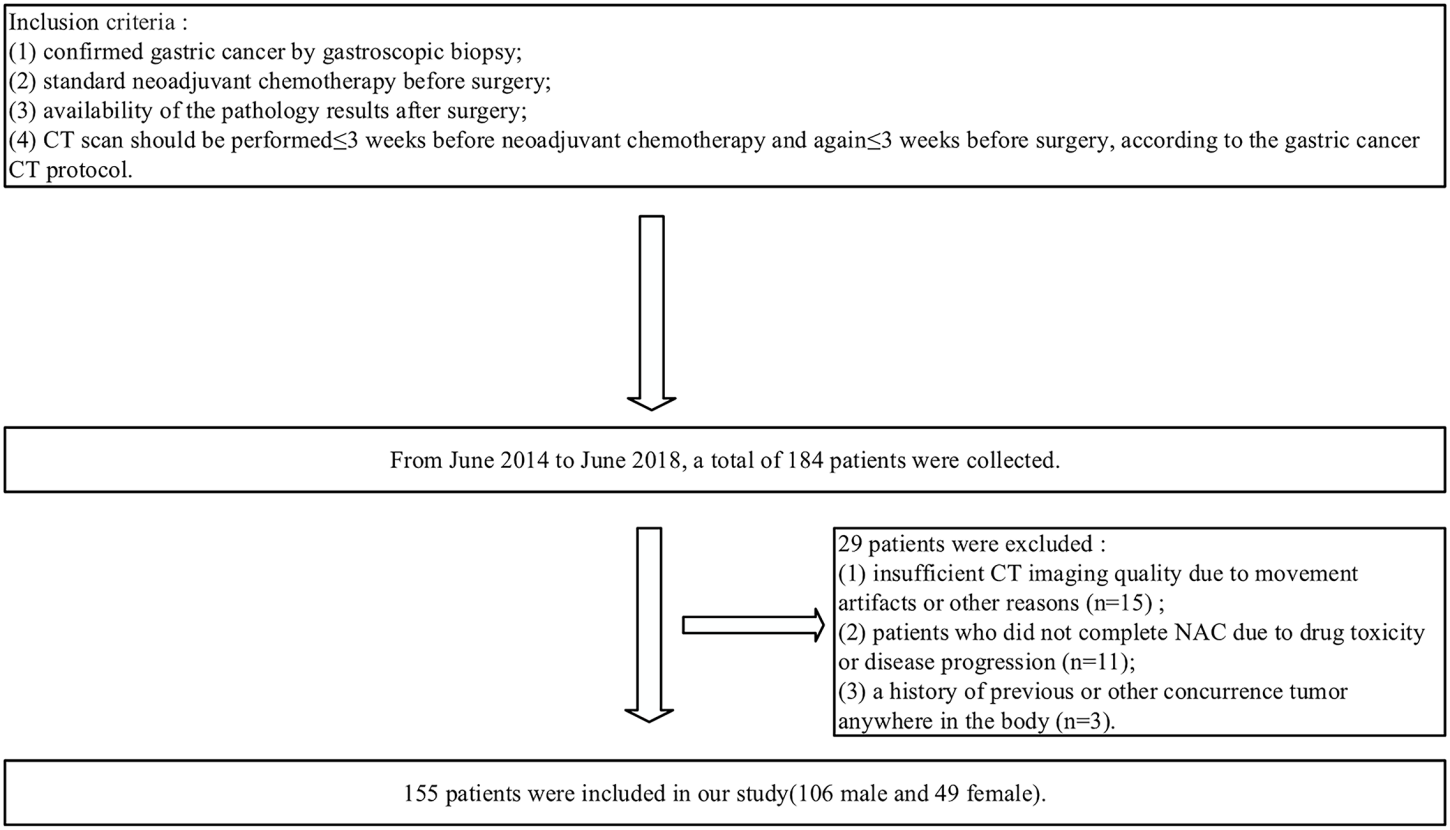
Flowchart of study enrollment.

A total of 184 patients were retrospectively enrolled from June 2014 to June 2018. The inclusion criteria were as follows: (1) confirmed gastric cancer by gastroscopic biopsy; (2) standard NAC before surgery; (3) availability of the pathology results after surgery; and (4) a visible tumor defined as cT2-4a/bNxM0 on CT images and CT scan performed ≤ 3 weeks before NAC and again ≤ 3 weeks before surgery, according to the gastric cancer CT protocol. The exclusion criteria were as follows: (1) insufficient CT imaging quality due to movement artifacts or other reasons (n=15); (2) any patients who did not complete NAC due to drug toxicity or disease progression (n=11); or (3) a history of previous or other concurrent tumor anywhere in the body (n=3). Ultimately, 155 patients were enrolled in our study (**Figure 1**). The median interval and interquartile range (IQR) between re-staging CT and surgery is 5 (IQR=2-6) days. The enrolled patients were randomly divided into a training cohort and testing cohort at the ratio of 7:3(110 and 45 patients), respectively, for model establishment and assessment.

### Neoadjuvant Chemotherapy

In our study, NAC for enrolled patients was administered according to the MAGIC study (24), which comprised three preoperative cycles of intravenous epirubicin (50 mg/m² of body-surface area) and cisplatin (60 mg/m²) on day 1, a continuous intravenous infusion of fluorouracil (200 mg/m²) for 21 days, and three postoperative cycles of the same regimen.

### Surgical and Pathological Evaluation

All the patients underwent gastrectomy with a standard D2 lymphadenectomy within three weeks after completion of chemotherapy (25, 26). Two pathologists (Ma QC and Yang CX) independently analyzed the surgical specimens for the invasion depth of the gastric wall (ypT staging) according to the pathologic TNM staging system developed by the American Joint Committee on Cancer and the International Union Against Cancer (25, 27). In addition, we also collected clinical factors including sex, location of the tumor, Borrmann type and tumor makers of all patients after chemotherapy, including alpha fetoprotein (AFP, normal reference value: <8.78ng/mL), carcinoembryonic antigen (CEA, normal reference value: <5ng/ml), carbohydrate antigen 125(CA 125, normal reference value: <35U/mL), carbohydrate antigen 724 (CA 724, normal reference value: <8.2U/mL), carbohydrate antigen (CA 199, normal reference value: <35U/mL) for univariate analysis.

### CT Image Acquisition

All gastric CT scans were performed using a third-generation dual-source scanner (SOMATOM Force; Siemens Healthineers, Forchheim, Germany) with the same scan protocol. All the patients were placed in a supine position on the scanner, and the parameters were set as follows (28): tube voltage A 90 kVp; effective tube current-time product 200mAs; tube voltage B 150 kVp; effective tube current-time product 125mAs; FOV: 374×374 mm; rotation time: 5 s; pitch: 0.6; kernel: Qr40; and collimation: 128*0.6 mm. All the patients were required to fast for 6 -8 h and drink 1000-1500ml of water before the CT scan. Using the test bolus technique, 16 mL of contrast agent, as a test bolus, was injected to monitor the time to reach the peak of the celiac trunk. Then, the main contrast agent (Ultravist; Schering, Berlin, Germany) was injected intravenously through the cubital vein at a flow rate of 3 ml/s (1.5 ml/kg body weight) using a CT-compatible power injector. Two phase-enhanced DECT scans were performed, including the arterial phase (at the beginning of the peak of the celiac trunk) and portal phase (delays 20 seconds after the peak of the celiac trunk).

All portal phase datasets were reconstructed with 1.5 mm slice thickness and delivered to a dedicated workstation with dual-energy software (Syngo.via, Version VB10, Siemens Healthineers, Forchheim, Germany) for further dual-energy image post processing. In addition, 120-kV equivalent mixed images were generated, linearly blended with a weighted factor of 0.6 (120 kVp), and the iodine maps (IM) were reconstructed and obtained from the dual-energy datasets (13, 29). The iodine map was based on the dual-energy, 3-material decomposition algorithm and represented the absolute iodine uptake value in the field of view (30). These two types of images were ultimately acquired for analysis.

### Image Analysis and Manual Classification

All 120 kVp CT images were independently analyzed by two radiologists experienced in gastrointestinal diseases (ZP and Ding B, both with more than 20 years of experience in the diagnosis of abdominal diseases). Both readers were partially blinded to the gastroscopic results (they knew that the patients had gastric cancer that had been diagnosed by endoscopic biopsy) and were completely unaware of the location, size, macroscopic features, and stage of gastric cancers. The inter-observer agreement of the two radiologists’ assessment of preoperative tumor restaging after NAC (ycT) were tested using weighted kappa statistics. The depth of tumor invasion judged by CT is based on the studies of Hasegawa and Habermann CR (31, 32). T1 tumors were defined as a tumor that cannot be seen on image or with focal thickening of the inner layer, visible in the outer layer of the gastric wall, and surrounded by a clear fat plane. T2 tumors were defined as localized or diffuse thickening of the gastric wall with transmural involvement and a smooth outer border of the wall or only a few small linear strands of soft tissue extending into the fat plane involving less than one-third of the tumor extent. T3 tumors were defined as transmural tumors with obvious blurring of at least one-third of the tumor extent or wide reticular strands surrounding the outer edge of the tumor. T4 tumors were defined as tumors in which the fat plane between the gastric tumor and the adjacent organs disappears or invaded the adjacent organs. T1, T2, and T3 tumors were defined as serosal invasion negative and T4 as serosal invasion positive.

All of the lesions were manually classified into a serosal invasion-negative group (−) and serosal invasion-positive group (+) by the radiologists on portal phase images. Finally, 57 cases of serosal invasion (−) and 98 cases of serosal invasion (+) were diagnosed.

### Tumor Segmentation and Feature Extraction

**Figure 2:**
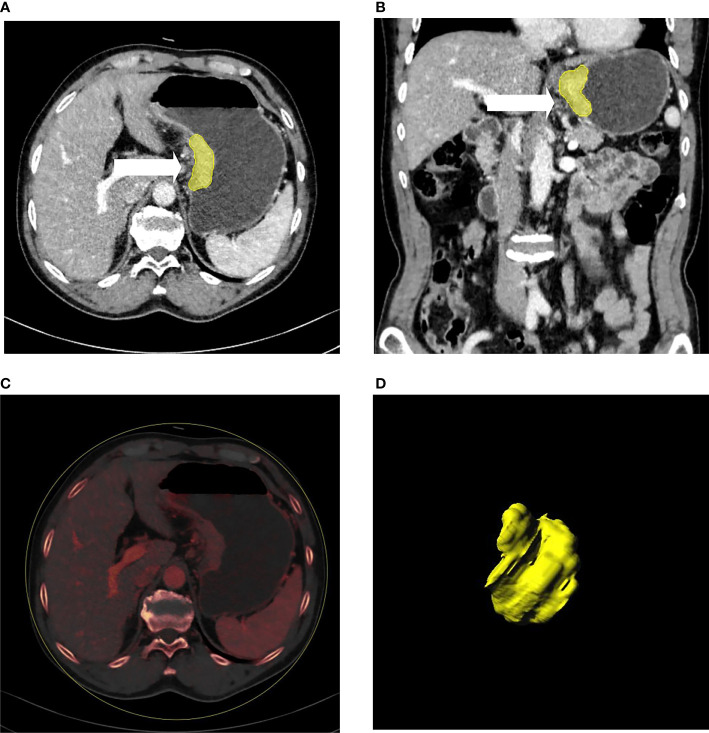
A patient with gastric cancer located in the cardia and lesser curve of the stomach **(A–D)**. Axial **(A)** and coronal **(B)** multiplanar reconstruction from portal phase images showed abnormal enhancement accompanied by wall thickening of the stomach. The irregular outer layer of the gastric wall, blurring, and reticular strands surrounding the outer border (arrow heads) indicated that this patient was serosal invasion-positive, which was proven by histology. **(C)** showed the iodine map of the lesion. 3D reconstruction of the lesion is displayed **(D)**.

Tumor segmentation and feature extraction were conducted with radiomics software (Radiomics 1.0.9a, Siemens Healthineers, Germany) on a research platform (Syngo.Via VB10, Research Frontier, Siemens Healthineers, Germany) (33). Two radiologists (Pan ZL and Du LJ) independently preformed tumor segmentation. Both of them were blinded to the pathological data but were informed that all the patients had gastric cancer. Volumes of interest (VOIs) were delineated semi-automatically in three dimensions on both 120 kVp images and were automatically matched to the IM images (**Figure 2**). Fat tissues or adjacent organs were excluded on coronal and sagittal panels. To ensure the consistency of the sketch between the two radiologists, 40 patients were randomly selected for secondary delineation.

The computation of radiomics features from VOIs of both 120 kVp and IM-120 kVp images was based on the PyRadiomics library (33). The extracted features were reproducible and matched the benchmarks of IBSI (34). In each set, there were 1,226 radiomics features extracted for each patient, including 234 first-order features, 17 shape features, and 975 texture features (texture features based on Gray Level Co-occurrence Matrix (GLCM) Features, Gray Level Size Zone Matrix (GLSZM) Features, Gray Level Run Length Matrix (GLRLM) Features, Gray Level Dependence Matrix (GLDM) Features, and Neighboring Gray Tone Difference Matrix (NGTDM) Features). A variety of options including Laplacian of Gaussian filtering, wavelet filtering, and non-linear intensity transforms including square, square root, logarithm and exponential, were provided by the software to customize image pre-processing before feature extraction.

### Feature Selection and Model Establishment

The feature stability and repeatability were initially evaluated. To reduce the influence in the manual segmentation, we calculated the consistency of all the extracted features (120 kVp and IM) by using Spearman’s rank (SR) correlation method. Setting the threshold of Spearman as 0.8, features with high consistency (SR > 0.8) were selected.

**Figure 3:**
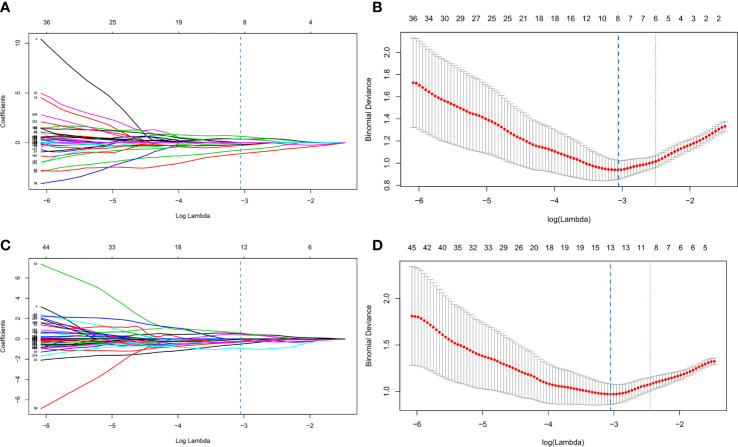
Process of least absolute shrinkage and selection operator (LASSO) logistic regression **(A–D)**. **(A, B)** represented LASSO logistic regression of 120 kVp features and **(C, D)** represented LASSO logistic regression of IM-120 kVp features. **(A, C)** showed LASSO coefficient profiles for the 1,226 features. The vertical line showed the optimal value of λ (λ=0.046 for 120 kVp, λ=0.0445 for IM-120 kVp) resulting in eight and thirteen non-zero features, respectively, for 120 kVp and IM-120 kVp. **(B, D)** showed that the area under the curve (AUC) curve was plotted by the tuning parameter (λ) selection performed by 10-fold cross-validation with the minimum deviance criterion.

After consistency analysis, least absolute shrinkage and selection operator (LASSO), which is appropriate for high-dimensional, low-sample size data with collinearity (35), was performed to screen out vital features for further analysis. Ten-fold cross-validation and minimum deviance information were used as the feature screening criteria. In our study, a multivariant logistic regression algorithm was applied as a classification model built with the remaining features. Based on these selected features with nonzero coefficients, two radiomics models were ultimately established: 120 kVp model (built with features extracted from 120 kVp images only) and IM-120 kVp model (built with features extracted from both 120 kVp and IM-120 kVp images). The process of LASSO is shown in **Figure 3**. In addition, a combination model (ComModel) was developed by adding independent preoperative predictors of serosal invasion from significant clinical characteristics for further evaluating the predictive value of dual-energy radiomics signatures.

### Model Performance and Comparison

**Figure 4:**
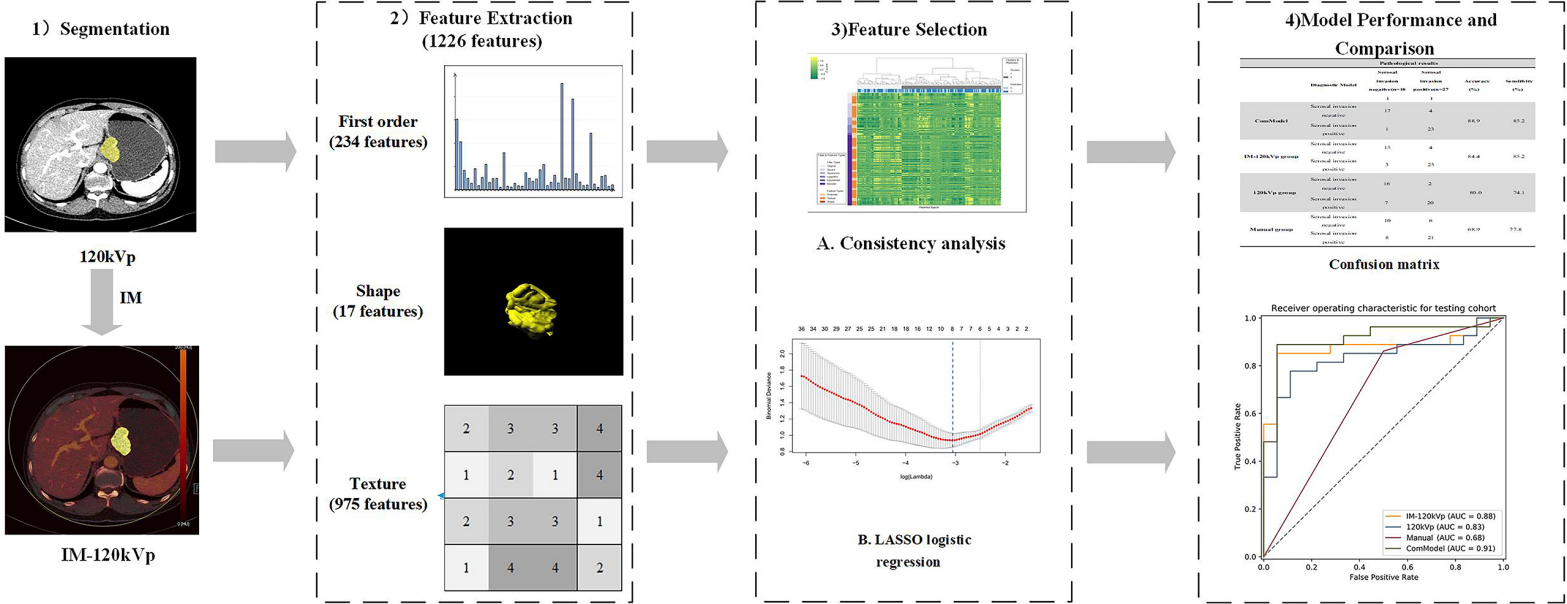
Flowchart of our study.

The performance of all prediction models was evaluated by the receiver operating characteristics (ROC) curve and area under the curve (AUC). The optimal thresholds of the odds for different models were determined by maximizing Youden’s J statistics. Sensitivity, specificity, accuracy, and the AUC were reported, as well as the 95% confidence intervals (CIs). The confusion matrix was also derived to illustrate the prediction ability. Furthermore, a diagnostic accuracy for detecting serosal invasion was calculated for all the models. In terms of the comparison of diagnostic efficiency among different models, DeLong’s test was conducted with significant differences set at p <0.05. Regarding to the goodness of fit of models, the calibration curve and Brier score were implemented for three regression models (120 kVp, IM-120 kVp and ComModel). Additionally, decision curve analysis (DCA) was performed for all diagnostic models to further assessing clinical gain. **Figure 4** shows the flowchart of our study.

### Statistical Analysis

Descriptive analysis was performed to describe the distribution of the variables of interest for the training and testing cohorts. The Kolmogorov-Smirnov test was used to test the normality of all the continuous variables. Student’s t-test or Wilcoxon rank sum test was used to compare normally or abnormally distributed continuous variables between the serosal invasion-positive and serosal invasion-negative groups, respectively. Categorical variables were compared between two groups using the chi-squared test or Fisher’s exact test, as appropriate. Inter-observer agreement was evaluated using the kappa coefficient. All tests were two-sided and p<0.05 was considered statistically significant. Feature selection, and model establishment and performance assessment were performed using the R software package (version 3.6.2). Other statistical analyses were implemented with SPSS (Version 25; IBM Corporation; Armonk, NY) and Medcalc Statistical Software (MedCalc Software, Ostend, Belgium; 2018).

## Results

### Clinical Characteristics of the Patients

**Table 1** describe the characteristics of the study cohort. The mean ages were 58.58 ± 10.12 and 59.66 ± 11.60 for training group and testing group, respectively. There was no bias for serosal invasion in the two groups (p = 0.595, *x* ^2^ = 0.282). Among all of the preoperative clinical factors, including sex, location of the tumor, Borrmann type and tumor markers were not significantly associated with serosal invasion after univariate analysis except ycT (both p<0.001, *x* ^2 =^ 19.563 and 32.308 for training group and testing group respectively), which was determined by radiologists as positive or negative.

**Table 1:** Characteristics of the patients.

Characteristic	Training Group (n=110)				Testing Group (n=45)				
	Total	Serosal invasion (−)(n=39)	Serosal invasion (+)(n=71)	p-value	Total	Serosal invasion (−)(n=18)		Serosal invasion (+)(n=27)	p-value
Age mean (SD), year	58.58(10.12)	59.13(10.63)	58.28(9.89)	0.677	59.66(11.60)		57.44(15.05)	59.10(11.12)	0.674
Sex, No.				0.800					0.357
Male	75	26	49		31		11	20	
Female	35	13	22		14		7	7	
Staging, No.				<0.001					<0.001
0	4	4	0		5		5	0	
I	14	13	1		9		9	0	
II	26	17	9		5		2	3	
III	66	5	61		26		2	24	
ycT, No.				<0.001					<0.001
1	5	5	0		5		5	0	
2	21	13	8		4		2	2	
3	11	3	8		7		4	3	
4	73	18	55		29		7	22	
Borrmann type				0.053					0.497
I	7	3	4		1		1	0	
II	50	15	35		17		6	11	
III	45	21	24		26		11	15	
IV	8	0	8		1		0	1	
Location, No.				0.723					0.540
Proximal	52	16	36		25		9	16	
Distal	58	23	35		20		9	11	
AFP				0.423					0.509
Positive (+)	12	3	9		2		0	2	
Negative (−)	98	36	62		43		18	25	
CEA				0.149					0.637
Positive (+)	19	4	15		10		4	6	
Negative (−)	91	35	56		35		14	21	
CA 125				0.656					0.400
Positive (+)	4	1	3		1		1	0	
Negative (−)	106	38	68		44		17	27	
CA 724				0.215					0.272
Positive (+)	21	5	16		10		2	8	
Negative (−)	89	34	55		35		16	19	
CA 19-9				0.705					0.557
Positive (+)	10	3	7		7		4	3	
Negative (−)	100	36	64		38		14	24	

SD, standard deviation.

### Radiomics Models Building and Validation

**Figure 5:**
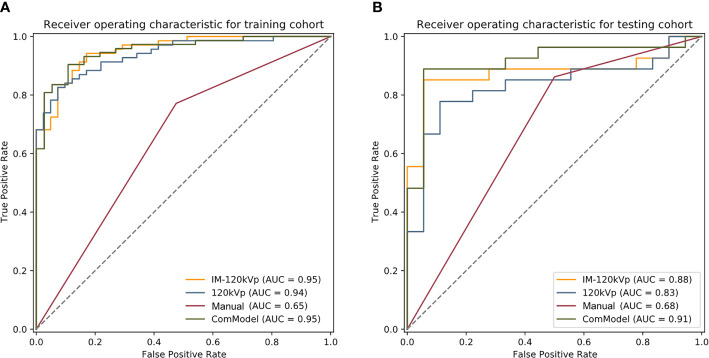
**(A, B)** Comparison among the four groups for the training and testing group, respectively.

After consistent analysis, 234 features from the 120 kVp group and 468 features from the IM-120 kVp group were selected. Based on this analysis, eight features were selected during LASSO from 120 kVp images (two first-order features, one shape feature, and five gray level features). Through the same process, 13 texture features (six from 120 kVp imaging and seven from IM-120 kVp imaging), three first-order features and 10 gray level features were selected for the IM-120 kVp model (**Supplementary 1**). The details of the selected features (boxplots and heatmaps) for two models are recorded in **Supplementary figures A–D**. Features contained in the models (and their coefficients) are shown in **Table 2**. Based on the 120 kVp images set, the model reached an AUC of 0.940 (95% CI: 0.8993–0.9805) in the training cohort (**Figure 5A**). The IM-120 kVp model revealed some improvement, with an AUC of 0.953 (95% CI: 0.9185–0.9875). In addition, a ComModel, developed with IM-120 kVp signature and ycT showed similar performance with an AUC of 0.953 (95% CI: 0.9173–0.9894). There was no significant difference between these three models (p=0.4 between IM-120 kVp model and 120kVp model, p=0.989 between ComModel and IM-120 kVp model, p=0.628 between ComModel and 120 kVp model). For the testing cohort, ComModel demonstrated a slightly better predictive performance for the detection of serosal invasion (AUC=0.914, 95% CI: 0.8219–1.000) than IM-120 kVp (AUC=0.879, 95% CI: 0.7685–0.9887, p=0.203) and a significant improvement in diagnose ability than 120 kVp (AUC=0.831, 95% CI: 0.7058–0.9568, p=0.018, **Figures 5A, B**). While IM-120 kVp (AUC=0.879, 95% CI: 0.7685–0.9887) model also showed significant better performance than 120 kVp (AUC=0.831, 95% CI: 0.7058–0.9568, p=0.040, **Figures 5A, B**) model in testing group.

**Table 2:** Features contained in the models and their coefficients.

	Feature	Coefficient
IM-120 kVp	Intercept	1.167599735
	M_original_firstorder_Entropy	0.5086991
	M_original_glcm_MCC	0.598577944
	M_squareroot_firstorder_10Percentile	−0.544459182
	M_logarithm_glrlm_RunEntropy	0.399207863
	M_wavelet.HHH_glcm_MaximumProbability	−0.348664531
	M_wavelet.LHL_gldm_DependenceVariance	−0.321889934
	IU_logarithm_glszm_LargeAreaHighGrayLevelEmphasis	−0.098944933
	IU_wavelet.HHH_glcm_MaximumProbability	−0.039030537
	IU_wavelet.HHL_glcm_MaximumProbability	−0.292194728
	IU_wavelet.HLL_glszm_SizeZoneNonUniformityNormalized	−0.245588102
	IU_wavelet.LLH_glrlm_RunVariance	−0.920372352
	IU_wavelet.LLH_glszm_LargeAreaHighGrayLevelEmphasis	−0.286325282
	IU_wavelet.LLL_firstorder_TotalEnergy	−0.037181896
120 kVp	Intercept	1.163715812
	M_original_firstorder_Entropy	0.359097918
	M_original_glcm_MCC	0.378549514
	M_original_shape_MinorAxisLength	−0.191729331
	M_squareroot_firstorder_10Percentile	−0.735779269
	M_logarithm_glrlm_RunEntropy	0.641373288
	M_wavelet.HHH_glcm_MaximumProbability	−1.107613619
	M_wavelet.LHL_gldm_DependenceVariance	−0.283627542
	M_wavelet.LHL_glszm_GrayLevelNonUniformity	−0.035049458

IM, Iodine map; GLCM, Gray Level Co-occurrence Matrix; GLSZM, Gray Level Size Zone Matrix; GLRLM, Gray Level Run Length Matrix; GLDM, Gray Level Dependence Matrix; NGTDM, Neighboring Gray Tone Difference Matrix.

### Performance Comparison Between the 120 kVp Model, IM-120 kVp Model, ComModel and Manual Classification

Regarding the manual classification, the k value for inter-observer agreement was 0.823 (95% CI: 0.732–0.913), which showed good agreement. The AUC of the classification was 0.648 (95%CI: 0.5555–0.7409) and 0.681 (95% CI: 0.5393–0.8228) for the training and testing cohorts, respectively. According to the calibration curve and Brier score, ComModel showed best goodness of fit than IM-120 kVp and 120 kVp groups (Brier score=0.081, 0.101 and 0.116, respectively for ComModel, IM-120 kVp and 120 kVp groups) (**Supplement Figure E**). Subsequently, we separately compared the AUC between the ComModel, IM-120 kVp, 120 kVp and manual groups. For the training cohort, the ComModel, IM-120 kVp, 120 kVp models showed significant differences in comparison with manual classification (p<0.001). For the testing cohort, the ComModel showed significant improvement than the manual group (p<0.001), the p value between the IM-120 kVp group and manual group was 0.034, whereas the 120 kVp group did not display superiority (p=0.124). In terms of the clinical gain, decision curve analysis illustrated that ComModel owned larger net benefit among the range of threshold probabilities compared with IM-120 kVp and 120 kVp models (**Supplement Figure F**). **Table 3** summarizes the accuracy, sensitivity, and specificity of the three models and manual classification of the testing group. The comparison among these four groups is shown in **Figures 5A, B**.

**Table 3:** Diagnostic performance of three models in testing group.

	Pathological results					
	Diagnostic Model	Serosal invasion negative(n=18)	Serosal invasion positive(n=27)	Accuracy (%)	Sensitivity (%)	Specificity (%)
**ComModel**	Serosal invasion negative	17	4	88.9	85.2	94.4
	Serosal invasion positive	1	23			
**IM-120 kVp group**	Serosal invasion negative	15	4	84.4	85.2	83.3
	Serosal invasion positive	3	23			
**120 kVp group**	Serosal invasion negative	16	7	80.0	74.1	88.9
	Serosal invasion positive	2	20			
**Manual group**	Serosal invasion negative	10	6	68.9	77.8	55.6
	Serosal invasion positive	8	21			

ComModel, Combine model; IM, Iodine map.

## Discussion

In this study, we developed and validated an IM-120 kVp radiomics model, which was superior to the radiomics model built by conventional 120 kVp, indicating the discrimination value of iodine from DECT for serosal invasion in GC patients after NAC. Furthermore, the ComModel model outperformed 120 kVp model and manual classification, presenting the incremental value in diagnosing serosal invasion. This, provides an assessment tool for treatment strategies for LAGC patients after NAC.

Accurate evaluation of serosal invasion for restaging of LAGC after NAC is critical, as it involves the choice of different interventions and the prognosis of patients. However, previous studies have found that the restaging is unreliable; the accuracy of T staging by CT was between 42.7% and 57% (11, 12). Because of the decreased number of tumor cells, edema, fibrosis, and chronic inflammation after chemotherapy (36), the blurred border of lesions seriously hinders accurate judgment. By extracting high-throughput quantitative imaging features, radiomics can characterize the spatial relationships and consistency of signal intensities within the tumor region (18), showing their utility in discriminating serosal invasion in preoperative staging (37, 38). In summary, our radiomics models performed well in accurately classifying serosal invasion not only in the training cohort, with AUCs of 0.940, 0.953 and 0.953 for the 120 kVp model, IM-120 kVp model and ComModel, respectively, but also in the testing cohort, with the AUCs of 0.831, 0.879 and 0.914, respectively. All revealed better performance than manual classification, especially for the ComModel. Through analysis and calculation of the extracted features, radiomics is helpful for finding small tumor tissue invisible to the naked eye, so it improves the accuracy of diagnosing serosal invasion after NAC. Furthermore, it also reflects the importance of clinical and radiological features in the judgment.

Another finding is that the IM-120 kVp radiomics model showed better discrimination than the 120 kVp model in restaging serosal invasion with LAGC after NAC. Iodine-specific maps have the potential to increase the depiction and characterization of hypoattenuating malignancies by increasing the contrast between a hypoattenuating lesion and normally enhancing parenchyma on the basis of differences in tissue iodine content (39). IU is a feasible biomarker with potential benefit not only in the anti-EGFR therapy response assessment for non-small cell lung cancer but also in predicting the radio-chemotherapy outcome for cervical cancer (40, 41). Chemotherapeutic agents used in NAC can decrease the capacity of the vascular bed and thus reduce the blood supply to tumor tissue (42). Iodine-containing contrast medium reaches tumor tissue *via* blood perfusion; thus, the iodine concentration in the tumor site can reflect the tumor response to chemotherapy. Both of the radiomics models displayed significant differences when compared with manual classification in the training group, and the model based on IM-120 kVp images also showed significant differences in the testing. Furthermore, iodine map images can slightly improve the accuracy of staging of gastric cancer compared with normal 120 kVp images, so the model based on IM-120 kVp images showed a better performance than the model based on 120 kVp images.

Our findings showed that entropy was closely related to identification of serosal invasion as it was included in both of the two models. Previous studies demonstrated that entropy was connected with a shorter survival time and was useful for risk stratification in gastric cancer and salivary gland carcinoma (43, 44). Heterogeneity is widely recognized as a feature of malignancy associated with cancer treatment failure and thus results in a poor prognosis (45–47). According to our results, many gray level features were screened out and included in our models, as well, inferring that gray level features can contribute to higher diagnostic accuracy. Though-run entropy (RE) was not mentioned in other studies, and their innate meanings were consistent with other gray level features in our model. The heterogeneity information of gastric cancer strongly indicated that intratumor heterogeneity is an essential factor in the restaging of ycT.

Our study had some limitations: First, this was a single-center study. Thus, multicenter validation in a larger sample size is needed to acquire high-level evidence for applying the model to clinical practice. Second, we only considered the restaging of ycT. Given that the restaging of lymph nodes was also an important factor for predicting prognosis, further studies should be designed to evaluate the iodine values of the regional lymph nodes. Third, we still adopt CT staging criteria because there are no studies to date that report on restaging criteria using CT in LAGC patients after NAC. Restaging criteria using CT after NAC for gastric cancer are urgently needed for critical decision-making.

NAC has been shown to significantly increase the curative resection rate, disease-free survival, and overall survival. The accurate assessment of clinical restaging, particularly the invasion of serosa after NAC, is critical for operative decision-making, so as to avoid potential toxicity, as well as to evaluate prognosis. Thus far, no modality has been accepted as an effective diagnostic method. Our dual-energy CT based radiomics models could help differentiate serosal invasion in preoperative restaging for LAGC. The radiomics features derived from IM show great potential in improving the capacity for diagnosis. In addition, a larger group of patient cohorts is needed to validate our models.

## Data Availability Statement

The raw data supporting the conclusions of this article will be made available by the authors, without undue reservation.

## Ethics Statement

The studies involving human participants were reviewed and approved by Ruijin Hospital Ethics Committee. The patients/participants provided their written informed consent to participate in this study.

## Author Contributions

The acquisition, data explanation, and manuscript draft were finished by LYW and YZ. ZP and BD are responsible for the analysis of CT images and the delineation of the VOIs. YC, JT, and LYW acquired the clinical information. JZ guided the chemotherapy regimen used. CY and QM analyzed and explained the pathological analysis. HZ and WY designed the study and made multiple revisions to the manuscript. All authors contributed to the article and approved the submitted version.

## Funding

This work was funded by the National Natural Science Foundation of China (81771789, 81771790), Shanghai Science and Technology Commission Science and Technology Innovation Action Clinical Innovation Field (18411953000) and Medical engineering cross research foundation of Shanghai Jiaotong University (YG2019ZDB09).

## Conflict of Interest

Authors YG and ZX were employed by the company Siemens Healthineers Ltd.

The remaining authors declare that the research was conducted in the absence of any commercial or financial relationships that could be construed as a potential conflict of interest.

## Abbreviations

AUC, Area under the curve; DECT, Dual-energy CT; EUS, Endoscopic ultrasound; GLCM, Gray Level Co-occurrence Matrix; GLDM, Gray Level Dependence Matrix; GLRLM, Gray Level Run Length Matrix; GLSZM, Gray Level Size Zone Matrix; IM, Iodine map; LAGC, Locally-advanced gastric cancer; LASSO, Least Absolute Shrinkage and Selection Operator; NAC, Neoadjuvant chemotherapy; NGTDM, Neighboring Gray Tone Difference Matrix; ROC, Receiver operating curve; VOI, Volume of interests.
