# Supplementary material for: The Performance of a Dual-Energy CT Derived Radiomics Model in Differentiating Serosal Invasion for Advanced Gastric Cancer Patients After Neoadjuvant Chemotherapy: Iodine Map Combined With 120-kV Equivalent Mixed Images
Source: Front Oncol. 2021 Jan 11;10:562945. doi: 10.3389/fonc.2020.562945 (PMC7874026; doi:10.3389/fonc.2020.562945)
Supplement: Supplementary file 1 [file DataSheet_1.docx]

**First order**

N_v_ represent the number of voxels included in the ROI

N_f_ represent the number of faces (triangles) defining the Mesh.

V the volume of the mesh in mm3, calculated by getMeshVolumeFeatureValue()

A the surface area of the mesh in mm2, calculated by getMeshSurfaceAreaFeatureValue()

Total Energy

$$total energy=V_{voxel}\sum_{i=1}^{Np} \left( X\left( i \right)+c \right)^{2}$$

Here, **c** is optional value, defined by voxelArrayShift, which shifts the intensities to prevent negative values in **X**. This ensures that voxels with the lowest gray values contribute the least to Energy, instead of voxels with gray level intensity closest to 0.

Total Energy is the value of Energy feature scaled by the volume of the voxel in cubic mm.

Entropy

$$entropy=-\sum_{i=1}^{N_{g}} p(i){log}_{2}\left( p\left( i \right)+\varepsilon\right)$$

Here, ε is an arbitrarily small positive number (≈2.2×10^−16^).

Entropy specifies the uncertainty/randomness in the image values. It measures the average amount of information required to encode the image values.

10th percentile

The 10^th^ percentile of **X**

**Shape**

In this group of features we included descriptors of the two-dimensional size and shape of the ROI. These features are independent from the gray level intensity distribution in the ROI and are therefore only calculated on the non-derived image and mask.

Unless otherwise specified, features are derived from the approximated shape defined by the circumference mesh. To build this mesh, vertices (points) are first defined as points halfway on an edge between a pixel included in the ROI and one outside the ROI. By connecting these vertices a mesh of connected lines is obtained, with each line defined by 2 adjacent vertices, which shares each a point with exactly one other line.

This mesh is generated using an adapted version marching cubes algorithm. In this algorithm, a 2x2 square is moved through the mask space (2d). For each position, the corners of the square are then marked ‘segmented’ (1) or ‘not segmented’ (0). Treating the corners as specific bits in a binary number, a unique square-index is obtained (0-15). This index is then used to determine which lines are present in the square, which are defined in a lookup table.

These lines are defined in such a way, that the normal of the triangle defined by these points and the origin is always oriented in a consistent direction. This results in signed values for the surface area of each triangle, so that when summed, the superfluous (postive) area included by triangles partly inside and outside the ROI is perfectly cancelled out by the (negative) area of triangles entirely outside the ROI.

Let:

$N_{p}$ represent the number of pixels included in the ROI

$N_{f}$ represent the number of lines defining the circumference (perimeter) Mesh.

***A*** the surface area of the mesh in mm2, calculated by getMeshSurfaceFeatureValue()

***P*** the perimeter of the mesh in mm, calculated by getPerimeterFeatureValue()

Minor Axis Length

$$minor axis=4\sqrt{\lambda_{minor}}$$

This feature yields the second-largest axis length of the ROI-enclosing ellipsoid and is calculated using the largest principal component λ_minor_.

The principal component analysis is performed using the physical coordinates of the pixel centers defining the ROI. It therefore takes spacing into account, but does not make use of the shape mesh.

**Gray Level Co-occurrence Matrix (GLCM) Features**

A Gray Level Co-occurrence Matrix (GLCM) of size $N_{g}\times N_{g}$ describes the second-order joint probability function of an image region constrained by the mask and is defined as $p(i,j\mid\delta,\theta)$. The ${(i,j)}^{th}$ element of this matrix represents the number of times the combination of levels $i$ and $j$ occur in two pixels in the image, that are separated by a distance of $\delta$ pixels along angle $\theta$. The distance $\delta$ from the center voxel is defined as the distance according to the infinity norm. For $\delta=1$, this results in 2 neighbors for each of 13 angles in 3D (26-connectivity) and for $\delta=2$a 98-connectivity (49 unique angles).

Let:

ε be an arbitrarily small positive number (≈2.2×10^−16^)

$\boldsymbol{P}(i,j)$ be the co-occurence matrix for an arbitrary $\delta$ and $\theta$

$p(i,j)$ be the normalized co-occurence matrix and equal to $\frac{\boldsymbol{P}(i,j)}{\sum\boldsymbol{P}(i,j)}$

$N_{g}$ be the number of discrete intensity levels in the image

$p_{x}\left( i \right)=\Sigma_{j=1}^{N_{g}}\boldsymbol{P}\left( i,j \right)$be the marginal row probabilities

$p_{y}\left( j \right)=\Sigma_{i=1}^{N_{g}}\boldsymbol{P}\left( i,j \right)$ be the marginal column probabilities

Maximal Correlation Coefficient (MCC)

$$MCC=\sqrt{second largest eigenvalue of Q}$$

$$Q\left( i,j \right)=\sum_{k=0}^{N_{g}} \frac{p(i,k)p(j,k)}{p_{x}(i)p_{y}(k)}$$

The Maximal Correlation Coefficient is a measure of complexity of the texture and 0≤MCC≤1.

In case of a flat region, each GLCM matrix has shape (1, 1), resulting in just 1 eigenvalue. In this case, an arbitrary value of 1 is returned.

Maximum Probability

$$maximum probability=max(p(i,j))$$

Maximum Probability is occurrences of the most predominant pair of neighboring intensity values.

**Gray Level Size Zone Matrix (GLSZM) Features**

A Gray Level Size Zone (GLSZM) quantifies gray level zones in an image. A gray level zone is defined as the number of connected voxels that share the same gray level intensity. A voxel is considered connected if the distance is 1 according to the infinity norm (26-connected region in a 3D, 8-connected region in 2D). In a gray level size zone matrix $\boldsymbol{P}(i,j)$ the ${(i,j)}^{th}$ element equals the number of zones with gray level $i$ and size $j$ appear in image. Contrary to GLCM and GLRLM, the GLSZM is rotation independent, with only one matrix calculated for all directions in the ROI.

Let:

$N_{g}$ be the number of discreet intensity values in the image

$N_{s}$ be the number of discreet zone sizes in the image

$N_{p}$ be the number of voxels in the image

$N_{z}$ be the number of zones in the ROI, which is equal to $\Sigma_{i=1}^{N_{g}}\Sigma_{j=1}^{N_{s}}\boldsymbol{P}\left( i,j \right)$ and 1≤$N_{z}$≤$N_{p}$

$\boldsymbol{P}\left( i,j \right)$ be the size zone matrix

$p\left( i,j \right)$be the normalized size zone matrix, defined as $p\left( i,j \right)=\frac{\boldsymbol{P(}i,j)}{N_{z}}$

Gray Level Non-Uniformity (GLN)

$$GLN=\frac{\Sigma_{i=1}^{N_{g}}{(\Sigma_{j=1}^{N_{s}}P(i,j))}^{2}}{N_{z}^{2}}$$

GLN measures the variability of gray-level intensity values in the image, with a lower value indicating more homogeneity in intensity values.

Size-Zone Non-Uniformity Normalized (SZNN)

$$SZNN=\frac{\Sigma_{j=1}^{N_{s}}{(\Sigma_{i=1}^{N_{g}}P(i,j))}^{2}}{N_{z}^{2}}$$

SZNN measures the variability of size zone volumes throughout the image, with a lower value indicating more homogeneity among zone size volumes in the image. This is the normalized version of the SZN formula.

Large Area High Gray Level Emphasis (LAHGLE)

$$LAHGLE=\frac{\Sigma_{i=1}^{N_{g}}\Sigma_{j=1}^{N_{s}}\frac{P(i,j)i^{2}}{j^{2}}}{N_{z}}$$

LAHGLE measures the proportion in the image of the joint distribution of larger size zones with higher gray-level values.

**Gray Level Run Length Matrix (GLRLM) Features**

A Gray Level Run Length Matrix (GLRLM) quantifies gray level runs, which are defined as the length in number of pixels, of consecutive pixels that have the same gray level value. In a gray level run length matrix $\boldsymbol{P}\left( i,j \right)\mid\theta$, the ${(i,j)}^{th}$ element describes the number of runs with gray level $i$ and length $j$ occur in the image (ROI) along angle $\theta$.

Let:

$N_{g}$ be the number of discreet intensity values in the image

$N_{r}$be the number of discreet run lengths in the image

$N_{p}$ be the number of voxels in the image

$N_{r}(\theta)$ be the number of runs in the image along angle $\theta$, which is equal to $\Sigma_{i=1}^{N_{g}}\Sigma_{j=1}^{N_{s}}\boldsymbol{P(}i,j\mid\theta)$ and 1≤$N_{r}(\theta)$≤$N_{p}$

$\boldsymbol{P(}i,j\boldsymbol{\mid}\theta)$ be the run length matrix for an arbitrary direction $\theta$

$p\boldsymbol{(}i,j\boldsymbol{\mid}\theta)$ be the normalized run length matrix, defined as $p\left( i,j \mid\theta\right)=\frac{\boldsymbol{P(}i,j\boldsymbol{\mid}\theta)}{N_{r}(\theta)}$

Run Variance (RV)

$${RV=\Sigma}_{i=1}^{N_{g}}\Sigma_{j=1}^{N_{r}}p(i,j\mid\theta){(j-\mu)}^{2}$$

Here,$\mu=\Sigma_{i=1}^{N_{g}}\Sigma_{j=1}^{N_{r}} p(i,j\mid\theta)j$

RV is a measure of the variance in runs for the run lengths.

Run Entropy (RE)

$${RE=-\Sigma}_{i=1}^{N_{g}}\Sigma_{j=1}^{N_{r}}p(i,j\mid\theta)\log_{2}( p\left( i,j \mid\theta\right)+\varepsilon)$$

Here, $\varepsilon$ is an arbitrarily small positive number (≈2.2×10^−16^).

RE measures the uncertainty/randomness in the distribution of run lengths and gray levels. A higher value indicates more heterogeneity in the texture patterns.

**Gray Level Dependence Matrix (GLDM) Features**

A Gray Level Dependence Matrix (GLDM) quantifies gray level dependencies in an image. A gray level dependency is defined as the number of connected voxels within distance δ that are dependent on the center voxel. A neighboring voxel with gray level $j$ is considered dependent on center voxel with gray level$i$ if |$i$ −$j$ |≤α. In a gray level dependence matrix $\boldsymbol{P(}i,j)$ the ${(i,j)}^{th}$ element describes the number of times a voxel with gray level $i$ with $j$ dependent voxels in its neighborhood appears in image.

Let:

$N_{g}$ be the number of discreet intensity values in the image

$N_{d}$ be the number of discreet dependency sizes in the image

$N_{z}$ be the number of dependency zones in the image, which is equal to $\Sigma_{i=1}^{N_{g}}\Sigma_{j=1}^{N_{d}}\boldsymbol{P(}i,j)$

$\boldsymbol{P(}i,j)$ be the dependence matrix

$p\left( i,j \right)$be the normalized dependence matrix, defined as $p(i,j)=\frac{\boldsymbol{P(}i,j)}{N_{z}}$

Dependence Variance (DV)

$${DV=\Sigma}_{i=1}^{N_{g}}\Sigma_{j=1}^{N_{d}}p(i,j){(j-\mu)}^{2}$$

Where$\mu=\Sigma_{i=1}^{N_{g}}\Sigma_{j=1}^{N_{d}} jp(i,j)$

Measures the variance in dependence size in the image.

**Supplement figure E.** The calibration curve and Brier score of the 120kVp, IM-120kVp and ComModel.

**Supplement figure F.** The decision curve of three models. The curve illustrated that ComModel owned larger net benefit among the range of threshold probabilities compared with IM-120kVp and 120kVp models.
