# Supplementary figures and images for: The Performance of a Dual-Energy CT Derived Radiomics Model in Differentiating Serosal Invasion for Advanced Gastric Cancer Patients After Neoadjuvant Chemotherapy: Iodine Map Combined With 120-kV Equivalent Mixed Images
Source: Front Oncol. 2021 Jan 11;10:562945. doi: 10.3389/fonc.2020.562945 (PMC7874026; doi:10.3389/fonc.2020.562945)

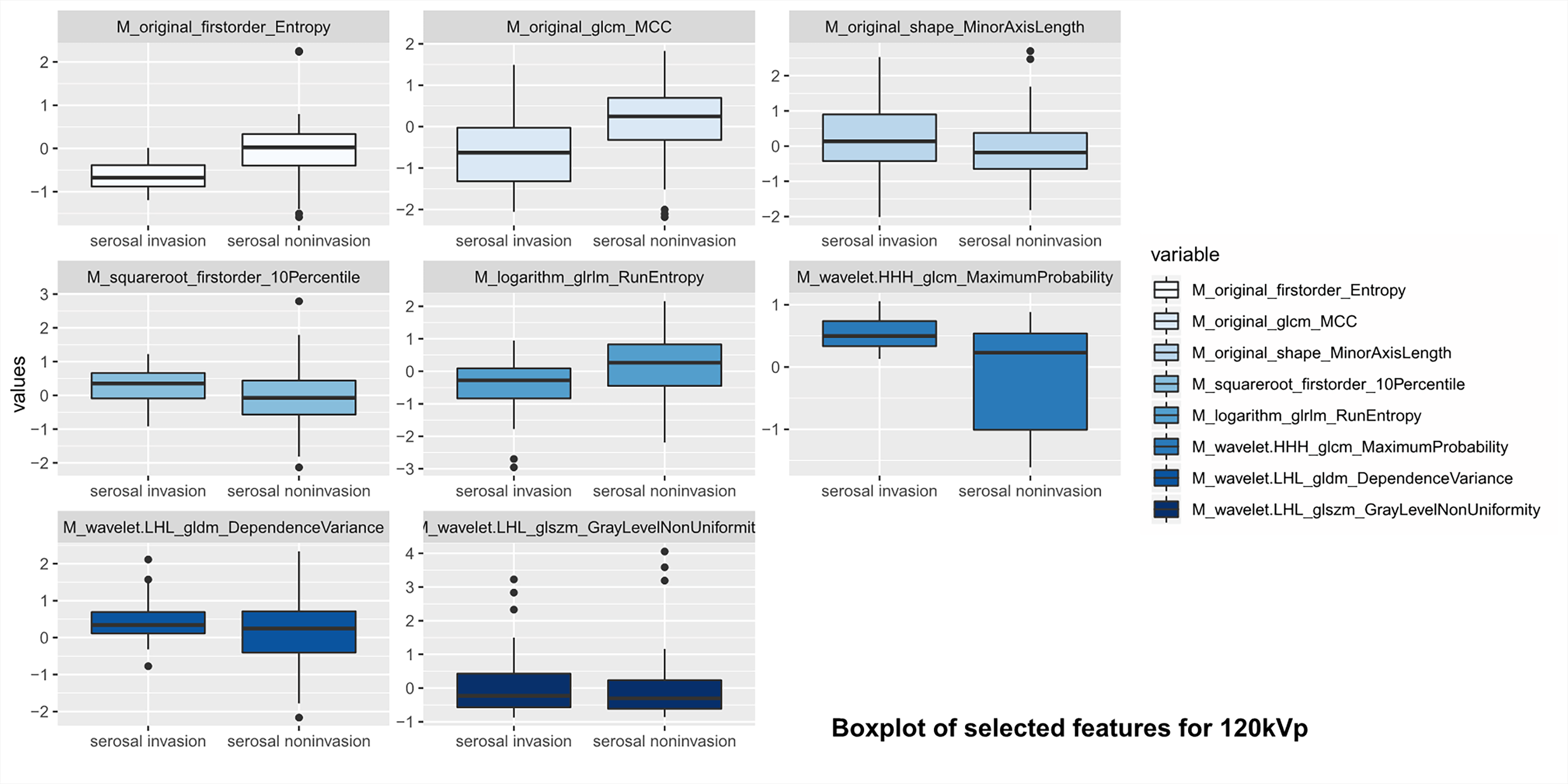

Supplement: Supplementary file 2 [file Image_1.tif]

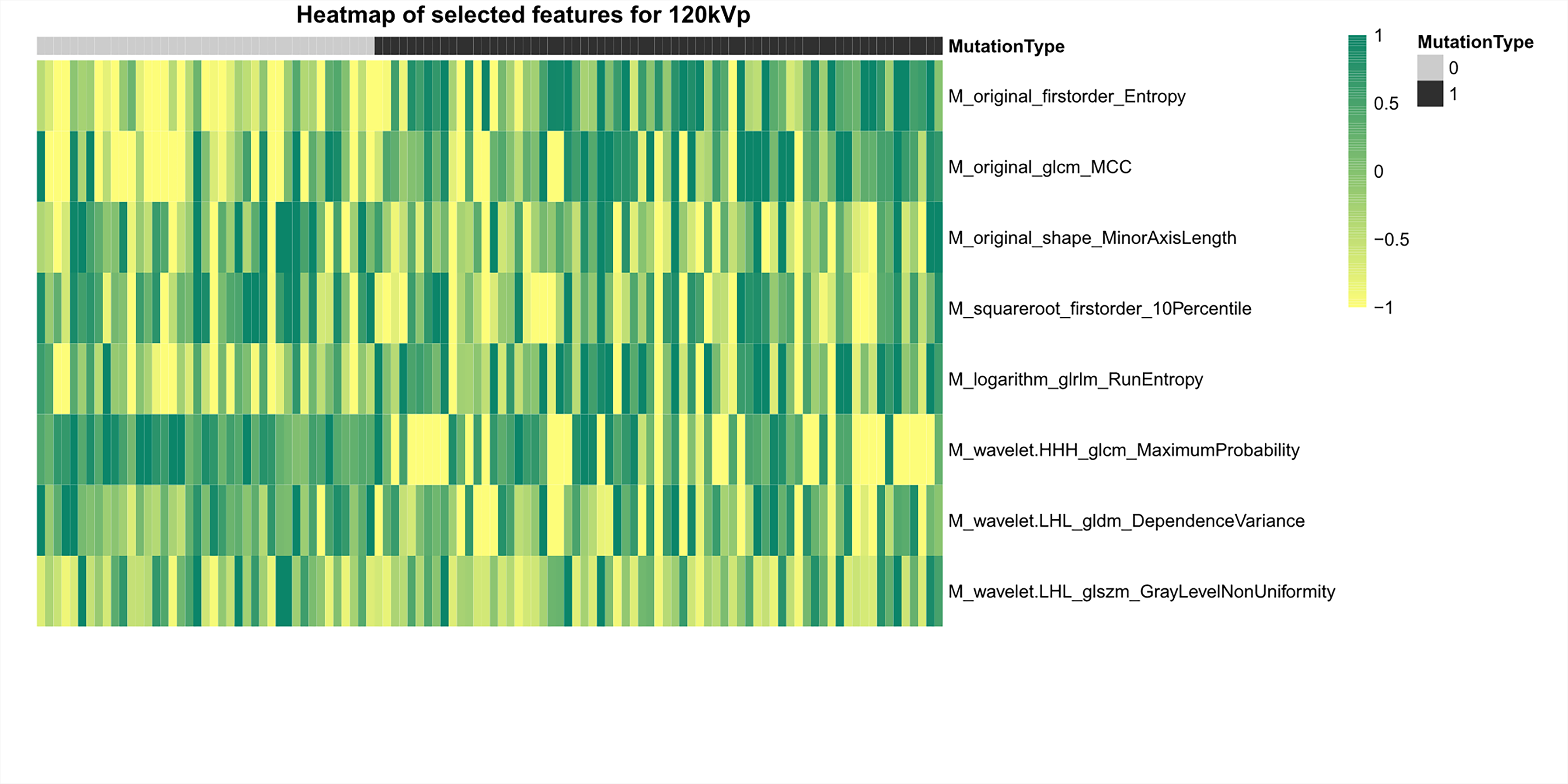

Supplement: Supplementary file 3 [file Image_2.tif]

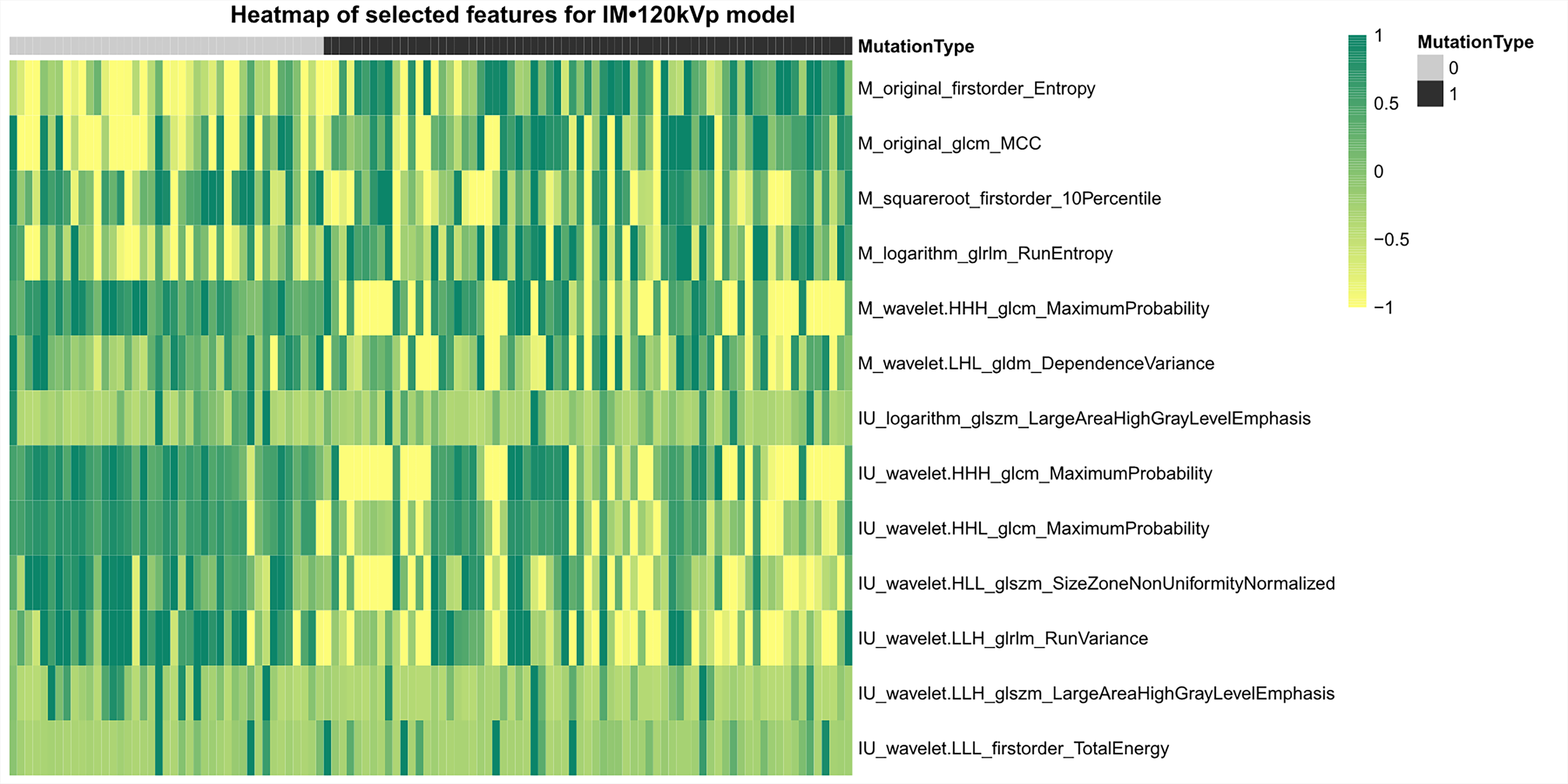

Supplement: Supplementary file 4 [file Image_3.tif]

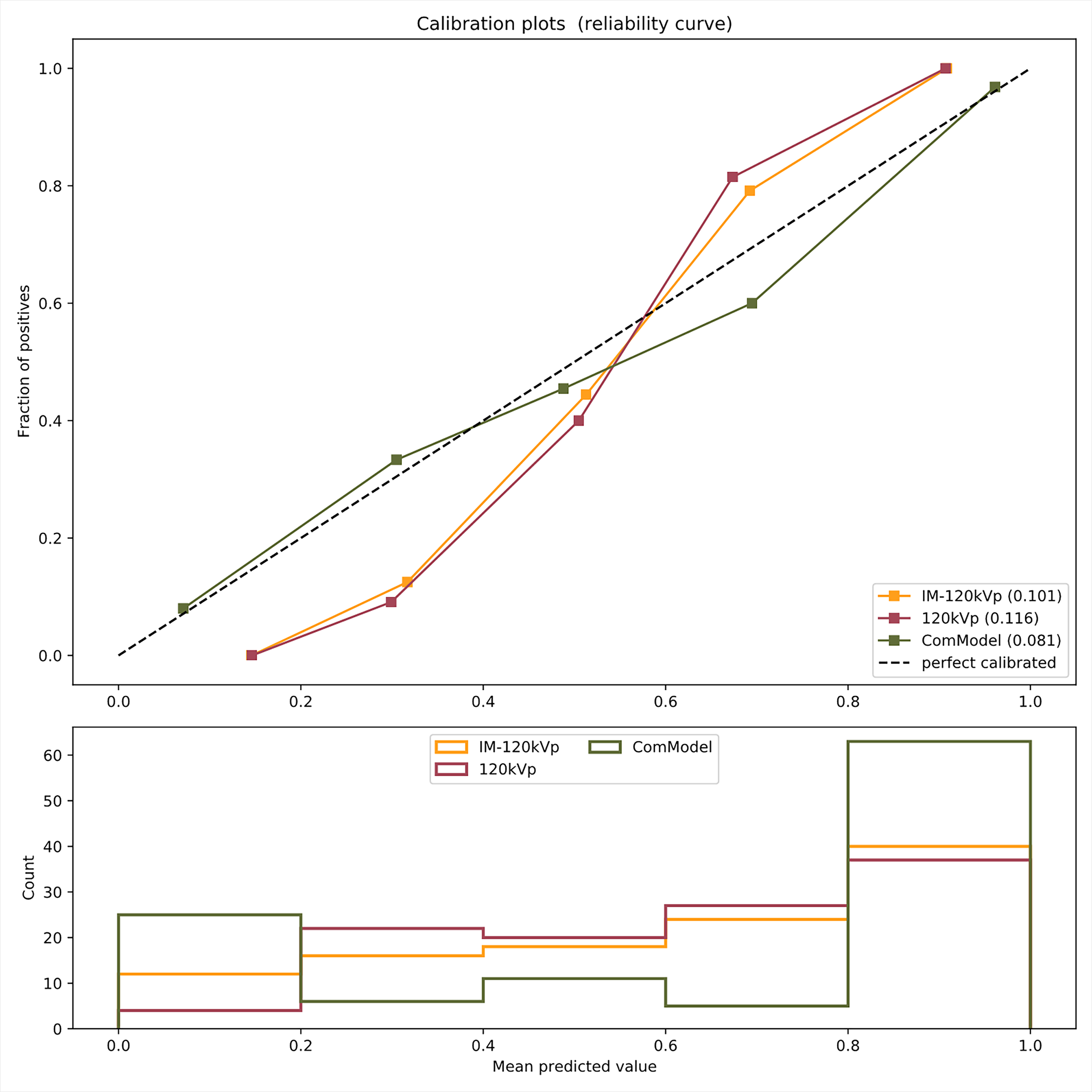

Supplement: Supplementary file 5 [file Image_4.tif]

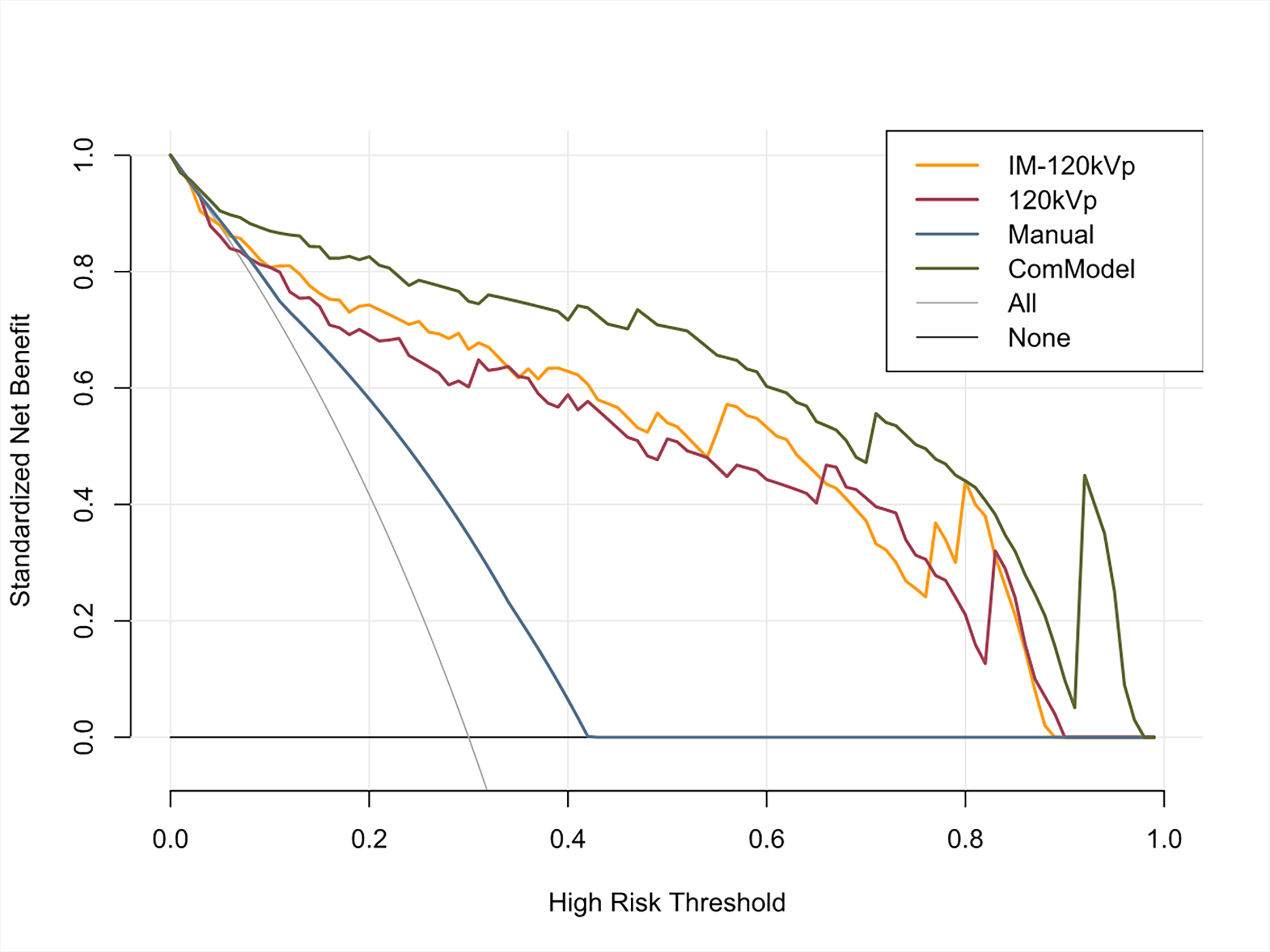

Supplement: Supplementary file 6 [file Image_5.tif]

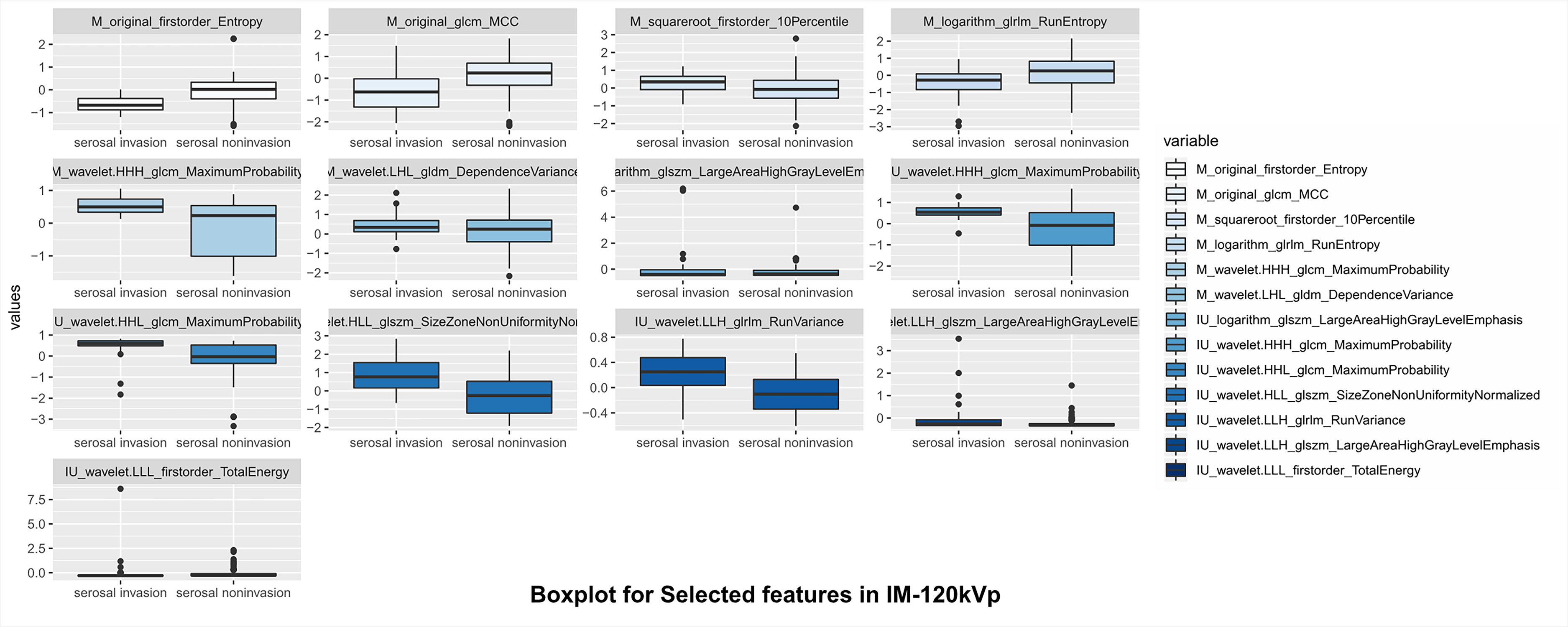

Supplement: Supplementary file 7 [file Image_6.tif]
